# A Smartphone-Based App to Improve Adjuvant Treatment Adherence to Multidisciplinary Decisions in Patients With Early-Stage Breast Cancer: Observational Study

**DOI:** 10.2196/27576

**Published:** 2021-09-16

**Authors:** Jing Yu, Jiayi Wu, Ou Huang, Xiaosong Chen, Kunwei Shen

**Affiliations:** 1 Department of General Surgery Comprehensive Breast Health Center Ruijin Hospital, Shanghai Jiao Tong University School of Medicine Shanghai China

**Keywords:** breast cancer, adherence, multidisciplinary treatment, adjuvant treatment, smartphone-based app, mobile phone

## Abstract

**Background:**

Multidisciplinary treatment (MDT) and adjuvant therapy are associated with improved survival rates in breast cancer. However, nonadherence to MDT decisions is common in patients. We developed a smartphone-based app that can facilitate the full-course management of patients after surgery.

**Objective:**

This study aims to investigate the influence factors of treatment nonadherence and to determine whether this smartphone-based app can improve the compliance rate with MDTs.

**Methods:**

Patients who had received a diagnosis of invasive breast cancer and had undergone MDT between March 2013 and May 2019 were included. Patients were classified into 3 groups: Pre-App cohort (November 2017, before the launch of the app); App nonused, cohort (after November 2017 but not using the app); and App used cohort (after November 2017 and using the app). Univariate and multivariate analyses were performed to identify the factors related to MDT adherence. Compliance with specific adjuvant treatments, including chemotherapy, radiotherapy, endocrine therapy, and targeted therapy, was also evaluated.

**Results:**

A total of 4475 patients were included, with Pre-App, App nonused, and App used cohorts comprising 2966 (66.28%), 861 (19.24%), and 648 (14.48%) patients, respectively. Overall, 15.53% (695/4475) patients did not receive MDT recommendations; the noncompliance rate ranged from 27.4% (75/273) in 2013 to 8.8% (44/500) in 2019. Multivariate analysis demonstrated that app use was independently associated with adherence to adjuvant treatment. Compared with the patients in the Pre-App cohort, patients in the App used cohort were less likely to deviate from MDT recommendations (odds ratio [OR] 0.61, 95% CI 0.43-0.87; *P*=.007); no significant difference was found in the App nonused cohort (*P*=.77). Moreover, app use decreased the noncompliance rate for adjuvant chemotherapy (OR 0.41, 95% CI 0.27-0.65; *P*<.001) and radiotherapy (OR 0.49, 95% CI 0.25-0.96; *P*=.04), but not for anti-HER2 therapy (*P*=.76) or endocrine therapy (*P*=.39).

**Conclusions:**

This smartphone-based app can increase MDT adherence in patients undergoing adjuvant therapy; this was more obvious for adjuvant chemotherapy and radiotherapy.

## Introduction

### Background

Breast cancer has the highest prevalence rate among malignant diseases in women worldwide [1]. Typically, comprehensive treatment for breast cancer includes locoregional and systemic therapy approaches. The former refers to surgery and radiotherapy, and the latter comprises chemotherapy, targeted therapy, endocrine therapy, and other promising strategies such as immunotherapy [2]. Moreover, multidisciplinary treatment (MDT), which involves physicians from different disciplines with specialized knowledge working as team to discuss the treatment of a given patient, has become a standard care modality that can promote clinical decision-making and improve the overall quality of treatment [3]. To date, nearly 70% to 80% of nonmetastatic breast cancers are curable because of the continuous improvement of therapeutic strategies and MDT discussions [4].

Despite the proven efficacy of adjuvant treatment, patients may have difficulty initiating or pursuing a treatment plan because of the relative complexity of the overall therapy. A cancer registry study demonstrated that the nonadherence rate for adjuvant therapy was approximately 30% for early breast cancer [5]. Meanwhile, it has been reported that nearly one-third of patients discontinued endocrine therapy during 5 years of treatment [6]. Noncompliance was associated with an increased risk of recurrence and poorer clinical outcomes [7,8]. Thus, it is essential to identify the reasons for nonadherence and use effective interventions to improve adherence.

### Mobile Health Intervention

Currently, the use of mobile devices to conduct health control and management has become increasingly popular and has the opportunity to affect health behaviors, especially in the condition of chronic disease and cancer setting among adolescents [9]. For example, sharing treatment experiences on the web can help alleviate isolation and emotional distress [10]. Active and regular self-monitoring using the digital app also helps to improve health-related outcomes, such as pain management for cancer [11]. Studies have also argued that the change in actual behavior is modest, and there is a lack of research focusing on effectiveness and acceptability [12,13]. New technologies such as artificial intelligence, virtual reality, and machine learning have also been tailored in health care settings to optimize disease outcomes [14]. Therefore, we launched a smartphone-based app called the *full-course management system* in November 2017, which enabled sustained communication between outpatients and medical workers and provided personalized management according to MDT discussions. Using this app, we aimed to facilitate the postoperative management of patients with breast cancer.

### Objective

In this study, we aimed to determine the potential factors that influence patient compliance with MDTs. Meanwhile, we aimed to evaluate whether this smartphone-based app could improve patients’ adherence to MDT for early breast cancer patients.

## Methods

### Data Processing and Cohort Sorting

Patients with a diagnosis of breast cancer who received MDT discussion for adjuvant treatment decisions between March 2013 and May 2019 at Shanghai Ruijin Hospital were retrospectively reviewed. Data on demographic and clinicopathological characteristics as well as follow-up information were retrieved from the Shanghai Jiao Tong University Breast Cancer Database. The inclusion criteria were as follows: (1) female patients with a diagnosis of primary breast cancer and (2) patients who received surgical treatment. Patients were excluded if they met the following criteria: (1) forgone or had missing MDT discussion data, (2) pathologically confirmed without invasive lesions, or (3) missing clinicopathological characteristic data. Patients were then divided into 3 cohorts according to the year of diagnosis and use of the smartphone-based management system. The Pre-App cohort included patients who received a diagnosis between March 2013 and October 2017. The App nonused cohort included patients who received a diagnosis between November 2017 and May 2019 without using the app. The App used cohort referred to patients who received a diagnosis between November 2017 and May 2019 and who had applied the smartphone-based app within the adjuvant treatment setting. It is worth noting that the use of the app was entirely up to patients’ willingness, and at least one time of logging record identified from the backstage system would be defined as app used.

### Ethics Approval and Consent to Participate

The study was approved by the Ethical Committees of Ruijin Hospital, Shanghai Jiao Tong University School of Medicine, and informed consent was waived because of the retrospective design of this study. All procedures performed in studies involving human participants were in accordance with the ethical standards of the institutional and national research committee and with the 1964 Helsinki declaration and its later amendments or comparable ethical standards.

### MDT Discussion

**Figure 1:**
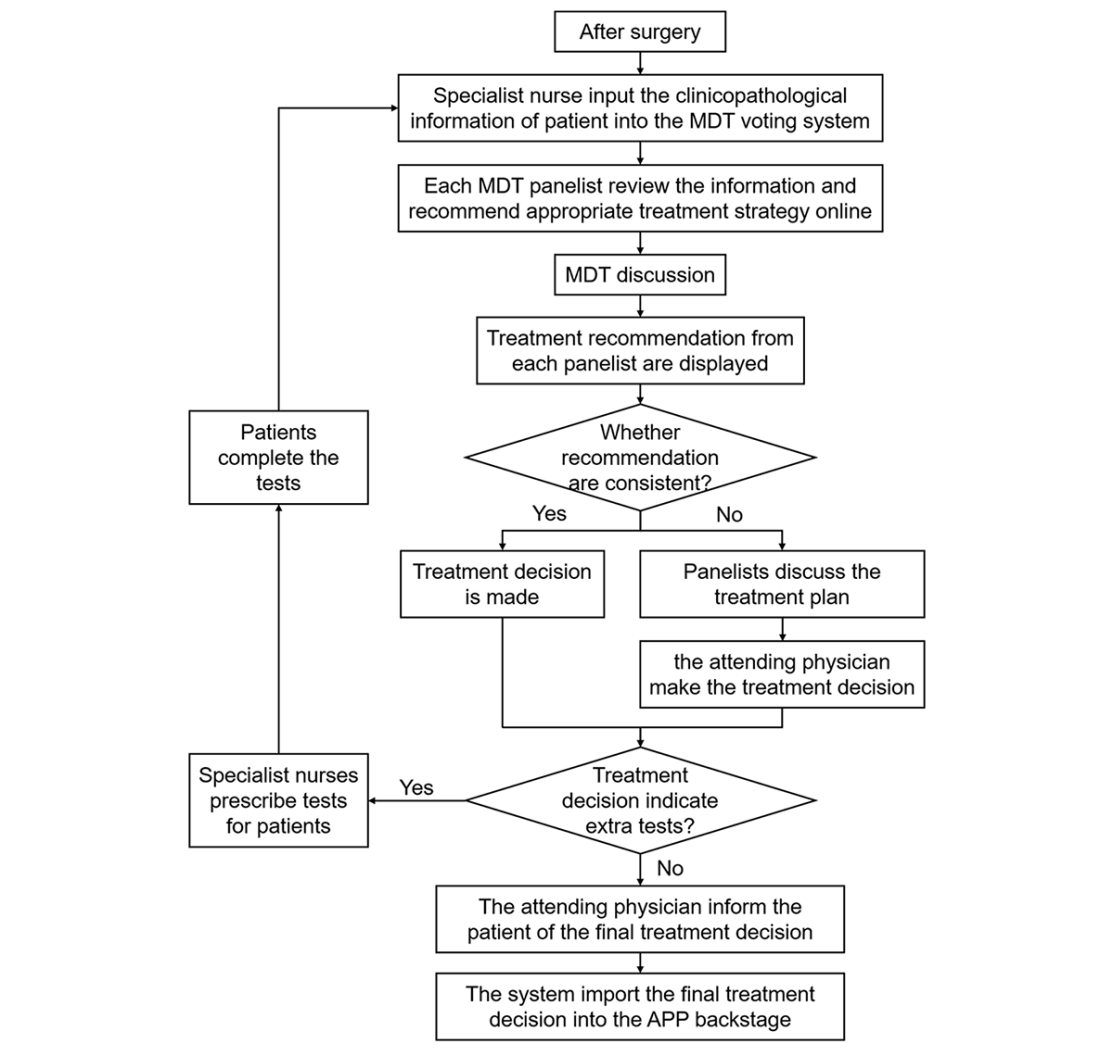
Flowchart of multidisciplinary treatment. MDT: multidisciplinary treatment.

After breast cancer surgery, an MDT discussion session was held to determine the adjuvant treatment strategy for each patient. In brief, the MDT panel members reviewed the medical history of each patient in advance and selected treatments on a given webpage. The MDT panelists consisted of breast surgeons, medical oncologists, pathologists, radiation oncologists, and oncology nurses. When the MDT meeting was held, a physician reported the cases and displayed the treatment results of each panelist for each patient. If the treatment results were consistent, a treatment decision was made. If the treatment decisions were inconsistent, the panel members discussed the treatment plan, and the attending physician made the final treatment decision for the patient. The flowchart of the MDT is shown in Figure 1.

### Smartphone-Based Full-Course Management App

The smartphone-based app consists of the following features:

1. Login, logout, setting, and modification of personal information
2. Full-course management included registering for information on the surgical treatment, viewing the results of the MDT discussion, generating the treatment process according to the MDT discussion, receiving the reminder of the treatment, confirming the completion of the treatment, filling in the follow-up information, and consulting a specialist nurse.
3. Questionnaire and feedback

**Figure 2:**
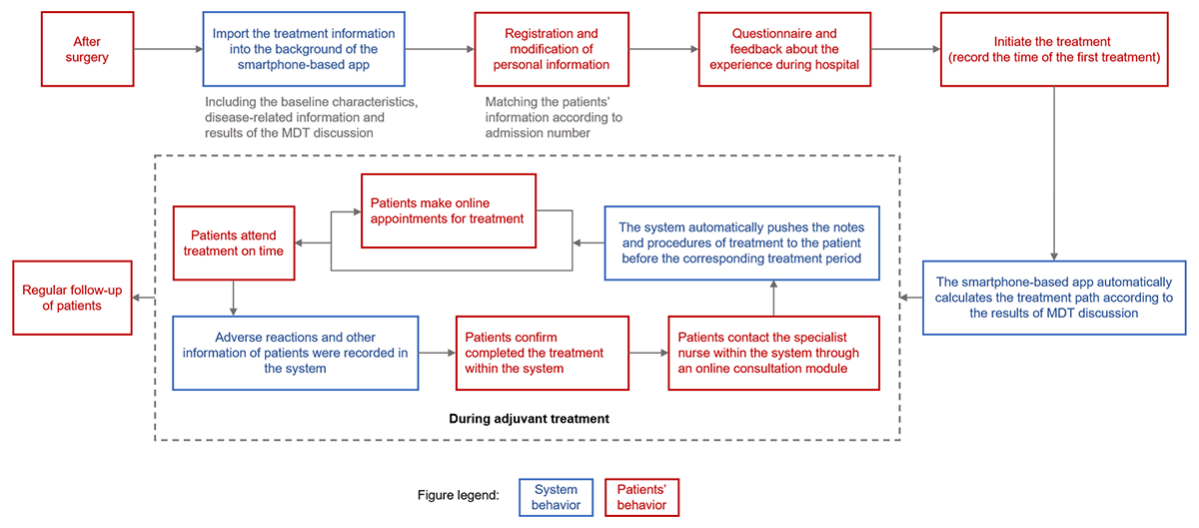
The operation flow of the smartphone-based app. MDT: multidisciplinary treatment.

The operational flow of the app is illustrated in Figure 2. In brief, after discharge from the hospital, the baseline characteristics and surgical and pathological information of the patients were uploaded to the system. Then, the patients completed the registration through the smartphone app and initiated the first course of treatment. The system then automatically calculated the treatment schedule according to the MDT recommendation and pushed notifications to patients periodically. After completing each cycle of therapy, patients were required to confirm the treatment-related record on the app. During the entire course of treatment, patients communicated in real time with specialist nurses via this app.

### Measurement of Nonadherence

Treatment adherence was assessed by physicians via outpatient procedures, by follow-up specialists, or by nurses via phone calls. The routine follow-up interval was as follows: (1) every 3 months within the first 2 years after surgery, (2) every 6 months between 3 and 5 years, and (3) once a year after 5 years.

Treatment adherence was evaluated in all patients according to whether their actual treatment was the same as the results of the MDT discussion. Nonadherence was defined as follows: (1) patients refused to receive the prescribed regimen recommended by the MDT, (2) patients received a different regimen with the MDT recommendation, or (3) patients failed to complete the full course of treatment. Adherence was investigated in each therapeutic modality, including chemotherapy, radiotherapy, anti-HER2 (human epidermal growth factor receptor-2) therapy, and endocrine therapy, and then overall compliance was calculated.

### Statistical Analysis

The chi-square test was used to evaluate the differences in patient characteristics among the three app-related groups and between the adherence and nonadherence groups, as well as to compare the difference in treatment adherence among the 3 app-related groups. A multivariate logistic regression model was used to identify the features associated with treatment compliance. Odds ratios (ORs) with 95% CIs were used to evaluate the influence of app use on patient adherence. Interactive analyses were performed to illustrate the effect of app use on compliance among patients with different clinicopathological features. Statistical analyses were performed using IBM SPSS version 25 and GraphPad Prism 8 (GraphPad Prism, Inc).

## Results

### Baseline Characteristics

**Figure 3:**
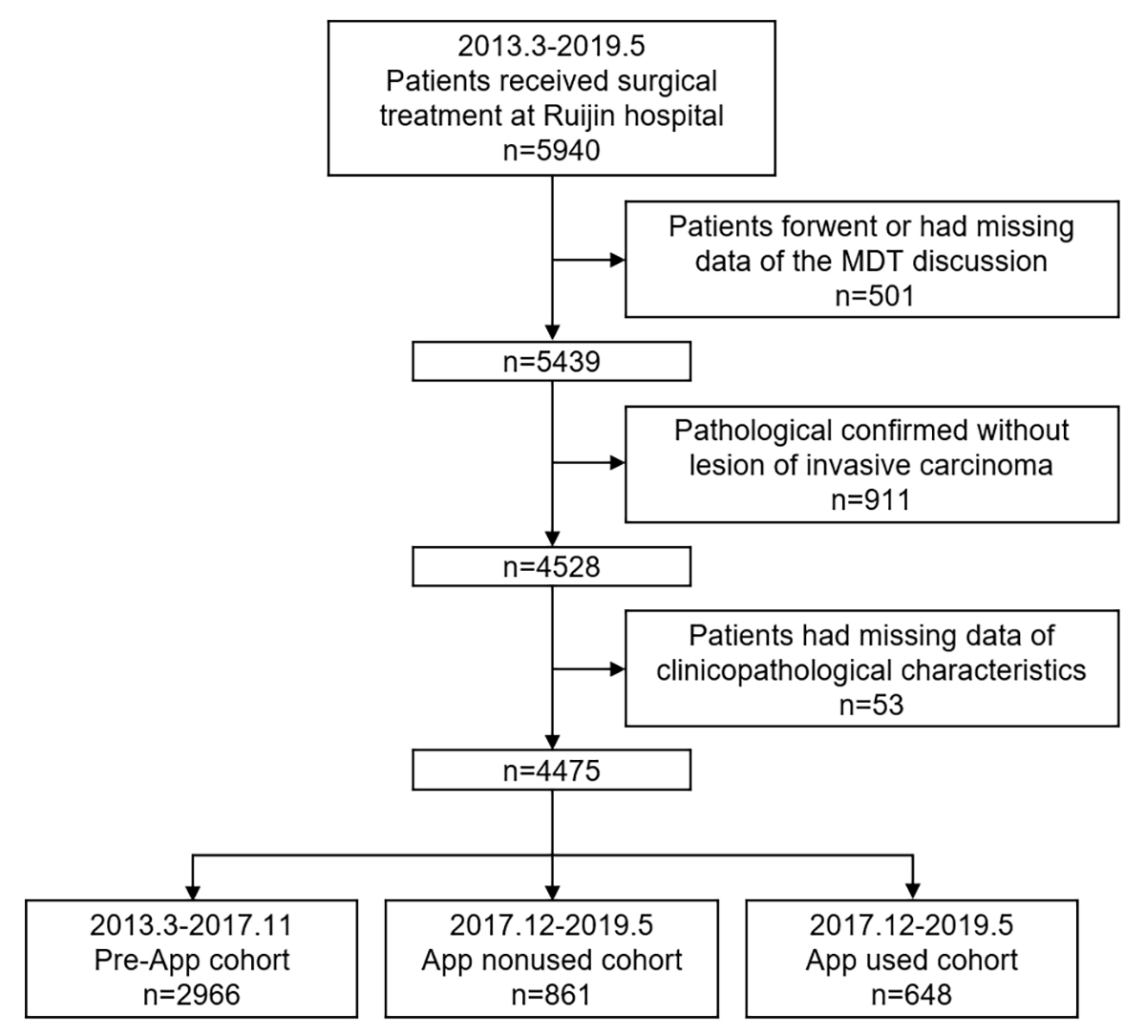
Flowchart. MDT: multidisciplinary treatment.

A total of 5940 patients were reviewed, and 4475 were included in the study (Figure 3). There were 66.28% (2966/4475), 19.24% (861/4475), and 14.48% (648/4475) of patients in the Pre-App, App nonused, and App used cohorts, respectively.

Among 4475 patients, 1662 (37.14%) were aged 50 years or older, 2304 (51.49%) were aged between 50 and 70 years, and 11.73% (509/4475) of patients were older than 70 years. A total of 64.31% (2878/4475) of patients underwent mastectomy for the breast, and 66.2% (2940/4475) of patients underwent sentinel lymph node biopsy for the axillary region. Regarding the molecular subtype, there were 20.63% (923/4475), 42.53% (1903/4475), 12.97% (540/4475), 11.17% (500/4475), and 13.61% (609/4475) of tumors classified as luminal-A like, luminal-B like (HER2-negative), luminal-B like (HER2-positive), HER2 positive, and triple negative, respectively (Table 1). When using the American Joint Committee on Cancer staging system to classify the disease, 44.71% (1954/4475) were stage I, 42.08% (1839/4475) were stage II, and 13.2% (577/4475) were stage III. Altogether, 84.46% (3780/4475) of patients received the treatment regimen assigned by MDT, which was defined as adherence.

**Table 1:** Baseline characteristics of patients who have received surgery and multidisciplinary treatment discussion (N=4475).

Characteristics		Values, n (%)					*P* value	
		Total (n=4475)	Pre-App cohort (n=2966)	App nonused cohort (n=861)	App used cohort (n=648)			
**Age (years)**								<.001
	≤50	1662 (37.1)	1100 (37.1)	293 (34)	269 (41.5)			
	50-70	2304 (51.5)	1535 (51.8)	435 (50.5)	334 (51.5)			
	>70	509 (11.4)	331 (11.2)	133 (15.4)	45 (6.9)			
**Educational level^a^**								.70
	Middle school or lower	1673 (38.2)	1104 (38.1)	334 (39.2)	235 (37.1)			
	High school or higher	2709 (61.8)	1792 (61.9)	518 (60.8)	399 (62.9)			
**Marital status**								.14
	Married	4315 (96.4)	2857 (96.3)	825 (95.8)	633 (97.7)			
	Others	160 (3.6)	109 (3.7)	36 (4.2)	15 (2.3)			
**Menopausal status**								.002
	Pre	1728 (38.6)	1144 (38.6)	300 (34.8)	284 (43.8)			
	Post	2747 (61.4)	1822 (61.4)	561 (65.2)	364 (56.2)			
**Benign breast disease history**								<.001
	Yes	967 (21.6)	572 (19.3)	228 (26.5)	167 (25.8)			
	No	3508 (78.4)	2394 (80.7)	633 (73.5)	481 (74.2)			
**Malignant disease history**								<.001
	Yes	209 (4.7)	111 (3.7)	56 (6.5)	42 (6.5)			
	No	4266 (95.3)	2855 (96.3)	805 (93.5)	606 (93.5)			
**Family history of breast cancer**								.002
	Yes	340 (7.6)	197 (6.6)	86 (10)	57 (8.8)			
	No	4135 (92.4)	2769 (93.4)	775 (90)	591 (91.2)			
**Comorbidity**								.01
	Yes	1766 (39.5)	1184 (39.9)	359 (41.7)	223 (34.4)			
	No	2709 (60.5)	1782 (60.1)	502 (58.3)	425 (65.6)			
**Breast surgery**								<.001
	Breast conserving	1597 (35.7)	1004 (33.9)	357 (41.5)	236 (36.4)			
	Mastectomy	2878 (64.3)	1962 (66.1)	504 (58.5)	412 (63.6)			
**Axillary surgery^b^**								<.001
	SLNB^c^	2940 (66.2)	1905 (64.6)	626 (74)	409 (63.2)			
	ALND^d^	1501 (33.8)	1043 (35.4)	220 (26)	238 (36.8)			
**Tumor size^e^**								.40
	≤2 cm	2542 (58)	1669 (58)	511 (59.3)	362 (55.9)			
	>2 cm	1843 (42)	1207 (42)	350 (40.7)	286 (44.1)			
**Lymph node status^f^**								<.001
	Negative	2905 (65.4)	1943 (65.9)	605 (71.5)	357 (55.3)			
	Positive	1535 (34.6)	1005 (34.1)	241 (28.5)	289 (44.7)			
**Pathological subtype**								<.001
	IDC^g^	3848 (86)	2578 (86.9)	690 (80.1)	580 (89.5)			
	Non-IDC	627 (14)	388 (13.1)	171 (19.9)	68 (10.5)			
**Tumor grade**								<.001
	I or II	2253 (50.3)	1437 (48.4)	469 (54.5)	347 (53.5)			
	III	1574 (35.2)	1137 (38.3)	212 (24.6)	225 (34.7)			
	Unknown	648 (14.5)	392 (13.2)	180 (20.9)	76 (11.7)			
**LVI^h^**								<.001
	No	3926 (87.7)	2689 (90.7)	722 (83.9)	515 (79.5)			
	Yes	549 (12.3)	277 (9.3)	139 (16.1)	133 (20.5)			
**Estrogen receptor status**								.01
	Negative	160 (24.7)	780 (26.3)	184 (21.4)	160 (24.7)			
	Positive	3351 (74.9)	2186 (73.7)	677 (78.6)	488 (75.3)			
**Progesterone receptor status**								<.001
	Negative	1629 (36.4)	1153 (38.9)	254 (29.5)	222 (34.3)			
	Positive	2846 (63.6)	1813 (61.1)	607 (70.5)	426 (65.7)			
**HER2^i^ status**								.001
	Negative	3435 (76.8)	2280 (76.9)	689 (80)	466 (71.9)			
	Positive	1040 (23.2)	686 (23.1)	172 (20)	182 (28.1)			
**Ki-67**								<.001
	<14%	1400 (31.3)	1029 (34.7)	245 (28.5)	126 (19.4)			
	≥14%	3075 (68.7)	1937 (65.3)	616 (71.5)	522 (80.6)			
**Molecular subtype**								<.001
	Luminal-A like	923 (20.6)	657 (22.2)	184 (21.4)	82 (12.7)			
	Luminal-B like (HER2-negative)	1903 (42.5)	1206 (40.7)	399 (46.3)	298 (46)			
	Luminal-B like (HER2-positive)	540 (12.1)	331 (11.2)	97 (11.3)	112 (10.8)			
	HER2 positive	500 (11.2)	355 (12)	75 (8.7)	70 (10.8)			
	Triple negative	609 (13.6)	417 (14.1)	106 (12.3)	86 (13.3)			
**TNM^j^ stage^k^**								.003
	Stage I	1954 (44.7)	1289 (44.8)	415 (49.1)	250 (38.7)			
	Stage II	1839 (42.1)	1213 (42.1)	329 (38.9)	297 (46)			
	Stage III	577 (13.2)	376 (13.1)	102 (12.1)	99 (15.3)			

^a^A total of 993 patients had missing data of educational level.

^b^A total of 34 patients had missing data of axillary surgery procedure.

^c^SLNB: sentinel lymph node biopsy.

^d^ALND: axillary lymph node dissection.

^e^A total of 90 patients had missing data of tumor size.

^f^A total of 35 patients had missing data of lymph node status.

^g^IDC: invasive ductal carcinoma.

^h^LVI: lymphovascular invasion.

^i^HER2: human epidermal growth factor receptor 2.

^j^TNM: tumor, lymph node, and metastasis.

^k^A total of 105 patients had missing data of tumor, lymph node, and metastasis stage.

### Factors Associated With Treatment Adherence

**Figure 4:**
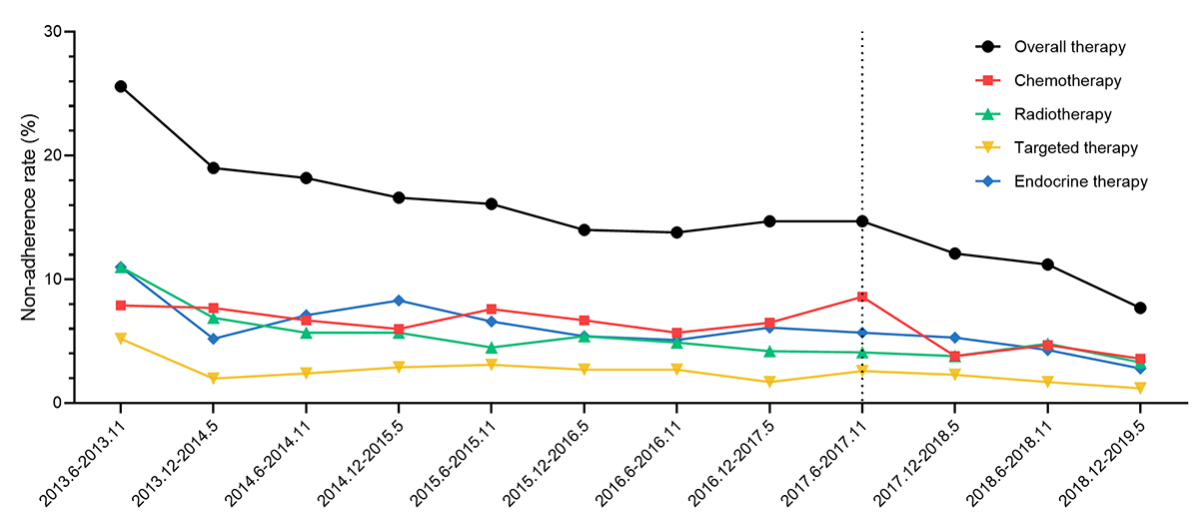
Trend of noncompliance rate to multidisciplinary treatment recommendations by year. The dotted line indicates launch of the smartphone-based app.

The rate of nonadherence decreased by year, which was approximately 27.4% in 2013 and 8.8% in 2019 (Figure 4).

In the univariate analysis, diagnosis year, demographic characteristics, medical history, surgical type, as well as the staging and characteristics of tumors were significantly associated with adherence to MDT. Moreover, regarding smartphone-based app use, the nonadherence rate was 17.6% in the Pre-App cohort, 13.24% in the App nonused cohort, and 9.1% in the App used cohort (*P*<.001; Tables 2 and 3; Table S1 in Multimedia Appendix 1).

**Table 2:** Factors associated with treatment adherence in patients.

Characteristics			Value, n (%)				*P* value
			Adherence (n=3780)		Nonadherence (n=695)		
**Year**							<.001
	2013.3-2014.5	410 (76.9)		123 (23.1)			
	2014.6-2015.5	453 (81.2)		105 (18.8)			
	2015.6-2016.5	551 (83.6)		108 (16.4)			
	2016.6-2017.5	677 (85.2)		118 (14.8)			
	2017.6-2018.5	799 (85.5)		136 (14.5)			
	2018.6-2019.5	890 (89.4)		105 (10.6)			
**Age (years)**							<.001
	≤50	1444 (86.9)		218 (13.1)			
	50-70	1972 (95.6)		332 (14.4)			
	>70	364 (71.5)		145 (28.5)			
**Educational level**							<.001
	Middle school or lower	1386 (81.8)		305 (18.2)			
	High school or higher	2332 (86.1)		377 (13.9)			
**Marital status**							.82
	Married	3646 (86.7)		669 (13.3)			
	Others	134 (83.1)		26 (16.9)			
**Menopausal status**							.001
	Pre	1498 (39.6)		230 (33.1)			
	Post	2282 (60.4)		465 (66.9)			
**Benign breast disease history**							.06
	Yes	836 (86.5)		131 (13.5)			
	No	2944 (83.9)		564 (16.1)			
**Malignant disease history**							.08
	Yes	167 (79.9)		42 (20.1)			
	No	3613 (84.7)		653 (15.3)			
**Family history of breast cancer**							.008
	Yes	304 (89.4)		36 (10.6)			
	No	3476 (84.1)		659 (15.9)			
**Comorbidity**							.08
	Yes	1471 (83.3)		295 (16.7)			
	No	2309 (85.2)		400 (14.8)			
**Breast surgery**							.32
	Breast conserving	1361 (85.2)		236 (14.8)			
	Mastectomy	2419 (84.1)		459 (15.9)			
**Axillary surgery**							.001
	SLNB^a^	2526 (67.2)		414 (60.7)			
	ALND^b^	1233 (32.8)		268 (39.3)			
**Tumor size (cm)**							.001
	≤2	2187 (86)		355 (14)			
	>2	1516 (82.3)		327 (47.9)			
**Lymph node status**							<.001
	Negative	2508 (86.3)		397 (13.7)			
	Positive	1250 (81.4)		285 (18.6)			
**Pathological subtype**							.007
	IDC^c^	3228 (83.9)		620 (16.1)			
	Non-IDC	552 (88)		75 (12)			
**Tumor grade**							<.001
	I or II	1938 (86)		315 (14)			
	III	1279 (81.3)		295 (18.7)			
	Unknown	563 (86.9)		85 (13.1)			
**LVI^d^**							.35
	No	3324 (84.7)		602 (15.3)			
	Yes	456 (83.1)		93 (16.9)			
**ER^e^ status**							.13
	Negative	933 (83)		191 (17.9)			
	Positive	2847 (85)		504 (15)			
**PR^f^ status**							.002
	Negative	1339 (82.2)		290 (17.8)			
	Positive	2441 (85.8)		405 (14.2)			
**HER2^g^ status**							<.001
	Negative	2941 (85.6)		494 (14.4)			
	Positive	839 (80.7)		201 (19.3)			
**Ki-67**							.001
	<14%	1219 (87.1)		181 (12.9)			
	≥14%	2561 (83.3)		514 (16.7)			
**Molecular subtype**							<.001
	Luminal-A like	831 (90)		92 (10)			
	Luminal-B like (HER2-negative)	1607 (84.4)		296 (15.6)			
	Luminal-B like (HER2-positive)	421 (78)		119 (22)			
	HER2 positive	418 (83.6)		82 (16.4)			
	Triple negative	503 (82.6)		106 (17.4)			
**TNM^h^ stage**							<.001
	Stage I	1713 (87.7)		241 (12.3)			
	Stage II	1512 (82.7)		318 (17.3)			
	Stage III	459 (79.5)		118 (20.5)			
**Group according to app use**							<.001
	Pre-App cohort	2444 (82.4)		522 (17.6)			
	App nonused cohort	747 (86.8)		114 (13.2)			
	App used cohort	589 (90.9)		59 (9.1)			

^a^SLNB: sentinel lymph node biopsy.

^b^ALND: axillary lymph node dissection.

^c^IDC: invasive ductal carcinoma.

^d^LVI: lymphovascular invasion.

^e^ER: estrogen receptor.

^f^PR: progesterone receptor.

^g^HER2: human epidermal growth factor receptor 2.

^h^TNM: tumor, lymph node, and metastasis.

**Table 3:** Patients’ adherence according to treatment.

Treatment			Value, n (%)							*P* value
			Pre-App cohort (n=2966)		App nonused cohort (n=861)		App used cohort (n=648)			
**Chemotherapy**										<.001
	Adherent	2732 (92.1)		808 (93.8)		625 (96.5)				
	Nonadherent	234 (7.9)		53 (6.2)		23 (3.5)				
**Radiotherapy**										.001
	Adherent	2789 (94)		809 (94)		633 (97.7)				
	Nonadherent	177 (6)		52 (6)		15 (2.3)				
**Targeted therapy^a^**										.01
	Adherent	594 (86.6)		158 (91.9)		170 (93.4)				
	Nonadherent	92 (13.4)		14 (8.1)		12 (6.6)				
**Endocrine therapy^b^**										<.001
	Adherent	2001 (91.2)		637 (93.5)		476 (96.4)				
	Nonadherent	194 (8.8)		44 (6.5)		18 (3.6)				
**Overall therapy**										<.001
	Adherent	2444 (82.4)		747 (86.8)		589 (90.9)				
	Nonadherent	522 (17.6)		114 (13.2)		59 (9.1)				

^a^In 1028 patients with HER2-positive breast cancer.

^b^In 3337 patients with hormone receptor–positive breast cancer.

Multivariate analysis demonstrated that diagnosis year, age, educational level, axillary surgery, lymph node status, HER2 status, and molecular subtype were significantly associated with patients’ adherence to the MDT recommendation. Notably, the use of the smartphone-based app was also an independent factor associated with patient compliance (*P*=.02). Compared with the patients in the Pre-App cohort, patients in the App used cohort were less likely to violate the treatment plan (OR 0.60, 95% CI 0.43-0.87; *P*=.007). However, there was no significant difference in adherence rates between the App nonused cohort and the Pre-App cohort (OR 0.96, 95% CI 0.70-1.29; *P*=.77; Table 4).

**Table 4:** Multivariate analysis of factors associated with nonadherence.

Characteristics			Odds ratio (95% CI)		*P* value	
Diagnosis year (per year)			0.88 (0.81-0.94)		<.001	
**Age (years)**						<.001
	50-70 vs ≤50	1.08 (0.88-1.31)		.45		
	>70 vs ≤50	2.85 (2.19-3.70)		<.001		
Educational level (high school or higher vs middle school or lower)			0.76 (0.64-0.91)		.002	
Menopausal status (post vs pre)			1.01 (0.72-1.41)		.96	
Family history of breast cancer (yes vs no)			0.70 (0.48-1.00)		.05	
Axillary surgery (ALND^a^ vs SLNB^b^)			0.72 (0.55-0.96)		.03	
Tumor size (>2 cm vs ≤2 cm)			0.83 (0.62-1.09)		.18	
Lymph node status (positive vs negative)			1.40 (1.04-1.88)		.03	
Pathological subtype (Non-IDC^c^ vs IDC)			0.77 (0.58-1.02)		.07	
**Tumor grade**						.15
	III versus I or II	1.19 (0.97-1.45)		.10		
	Unknown versus I or II	1.36 (0.84-2.20)		.21		
PR^d^ status (positive vs negative)			0.86 (0.66-1.12)		.28	
HER2^e^ status (positive vs negative)			2.04 (1.34-3.10)		.001	
Ki-67 index (≥14% vs <14%)			0.96 (0.73-1.26)		.75	
**Molecular subtype**						<.001
	Luminal-B like (HER2 negative) versus Luminal-A like	1.83 (1.29-2.59)		.001		
	Luminal-B like (HER2 positive) versus Luminal-A like	1.60 (1.16-2.21)		.005		
	HER2 positive versus Luminal-A like	N/A^f^		N/A		
	Triple negative versus Luminal-A like	2.11 (1.41-3.14)		<.001		
**TNM^g^ stage**						.02
	Stage II versus stage I	1.35 (1.08-1.69)		.008		
	Stage III versus stage I	1.57 (1.09-2.25)		.01		
**Group according to app use**						.02
	App nonused cohort versus Pre-App cohort	0.96 (0.70-1.29)		.77		
	App used cohort versus Pre-App cohort	0.61 (0.43-0.87)		.007		

^a^ALND: axillary lymph node dissection.

^b^SLNB: sentinel lymph node biopsy.

^c^IDC: invasive ductal carcinoma.

^d^PR: progesterone receptor.

^e^HER2: human epidermal growth factor receptor 2.

^f^N/A: not applicable.

^g^TNM: tumor, lymph node, and metastasis.

### Patient Adherence According to Treatment

#### Adjuvant Chemotherapy

Among 4475 patients, 310 (6.93%) patients did not adhere to the adjuvant chemotherapy decision, and the nonadherence rates were 7.9%, 6.2%, and 3.5% in the Pre-App cohort, App nonused, and App used cohorts, respectively (*P*<.001; Table 3; Table S1 in Multimedia Appendix 1). In the multivariate analysis, older age, luminal-B–like tumors, HER2-positive breast cancer, and triple-negative breast cancer were significantly associated with a lower compliance rate. Pathologically diagnosed noninvasive ductal carcinoma was associated with good compliance (*P*=.003). Moreover, compared with the patients in the Pre-App cohort, patients in the App used cohort (OR 0.41, 95% CI 0.27-0.65; *P*<.001) but not in the App nonused cohort (OR 0.74, 95% CI 0.54-1.02; *P*=.07) were less likely to not follow MDT-recommended chemotherapy (Table S2 in Multimedia Appendix 1).

#### Adjuvant Radiotherapy

There were 5.45% (244/4475) patients who did not receive adjuvant radiotherapy according to the MDT discussion, and the discordance rates were 6%, 6%, and 2.3% in the 3 groups, respectively (*P*=.001; Table 3; Table S3 in Multimedia Appendix 1). Multivariate analysis demonstrated that later diagnosis year, age, educational level, Ki-67 index, and lymph node status were significantly related to patient adherence (Table S4 in Multimedia Appendix 1). When compared with the patients in the Pre-App cohort, patients in the App used cohort were less likely to not follow the MDT recommendation (OR 0.49, 95% CI 0.25-0.96; *P*=.004). However, there was no significant difference between the App nonused cohort and the Pre-App cohort (OR 1.38, 95% CI 0.86-2.22; *P*=.18) in terms of adjuvant radiotherapy.

#### Adjuvant Target Therapy

Among 1028 patients with HER2-positive breast cancer, 118 (11.48%) patients violated the anti-HER2 treatment strategy. The nonadherence rates were 13.4%, 8.1%, and 6.6% in the Pre-App, App nonused, and App used cohorts, respectively (*P*=.02; Table 3; Table S5 in Multimedia Appendix 1). In multivariate analysis, only diagnosis year, age >70 years, and educational level were independent factors of treatment adherence (Table S6 in Multimedia Appendix 1). However, app use was not independently associated with MDT adherence to adjuvant targeted therapy (*P*=.76).

#### Adjuvant Endocrine Therapy

A total of 3337 patients received a diagnosis of hormone receptor–positive breast cancer, among whom 256 (7.67%) did not follow the doctors’ recommendations for endocrine therapy within the follow-up period. The noncompliance rates were 8.8%, 6.5%, and 3.6% in the 3 groups (*P*<.001; Table 3; Table S7 in Multimedia Appendix 1). Multivariate analysis revealed that patients with early diagnosis years, aged >70 years, and with luminal-B–like (HER2-positive) breast cancer were less likely to adhere to the endocrine therapy recommendation (Table S8 in Multimedia Appendix 1). The use of the app was not significantly associated with treatment compliance (*P*=.54).

### Subgroup Analysis

**Figure 5:**
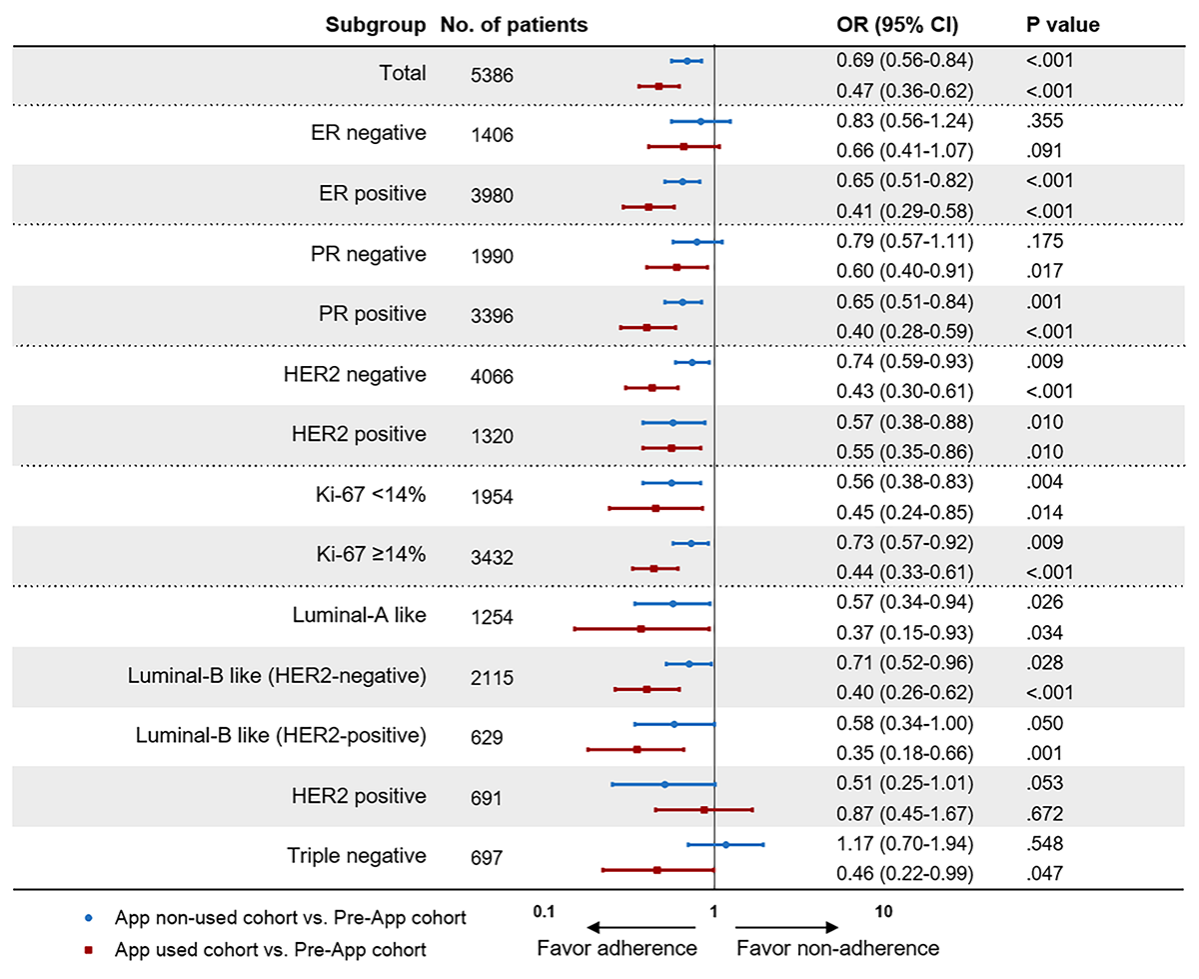
Effect of the app use on multidisciplinary treatment compliance in the entire population and stratified according to clinicopathological features. Odds ratios (OR) and 95% CIs for multidisciplinary treatment compliance (an OR<1 indicates a higher compliance rate) are shown. The compliance rate of patients in the Pre-App cohort was used as a reference. CIs have not been adjusted for multiple comparisons. Significant interactions were observed between patient groups and age, menopausal status, as well as comorbidities. ER: estrogen receptor; HER2: human epidermal growth factor receptor-2; OR: odds ratio; PR: progesterone receptor.

Subgroup analyses of MDT-recommended treatment compliance according to clinicopathological parameters were also performed (Figure 5). Age (interaction *P*<.001), menopausal status (interaction *P*<.001), and comorbidity (interaction *P*=.007) were significantly associated with app use and MDT compliance. In patients older than 70 years, those who did not use the app were more likely to be nonadherent (OR 2.01, 95% CI 1.31-3.09; *P*<.001). Meanwhile, use of apps was associated with better adherence in postmenopausal patients (OR 0.54, 95% CI 0.38-0.77; *P*<.001) and patients with comorbidities (OR 0.46, 95% CI 0.28-0.74; *P*<.001). However, there were no significant interactions between the group setting and cancer-related characteristics, including the molecular subtype or tumor, lymph node, and metastasis stage.

## Discussion

### Principal Findings

In this study, we included 4475 patients with early-stage breast cancer who had undergone an MDT discussion after surgery and found that sociodemographic factors together with clinicopathological factors were associated with MDT compliance in terms of adjuvant treatments. More importantly, the novel smartphone-based app designed for full-course management of patients with breast cancer could improve patients’ adherence to MDT recommendations for adjuvant treatment, which will help us better manage breast cancer adjuvant treatment after MDT and improve disease outcomes for these patients.

### MDT in Breast Cancer

Currently, MDT has gradually become an integral part of standardized treatment modalities for breast cancer worldwide. It could be conducive to evidence-based decisions for clinicians and provide a convenient medical treatment process for patients [15,16]. Our previous studies demonstrated that MDT discussion could also lower the relative risk of relapse by 16% and the risk of death by 11% in patients with breast cancer [7]. However, a major difficulty after the MDT meeting and during the treatment process was maintaining patient compliance. Data from the Pennsylvania and Florida cancer registries demonstrated that nearly 30% of the patients did not pursue the recommended treatment. The reported nonadherence rate during the 5 years of adjuvant endocrine therapy ranged from 10% to 40% [17-20]. Our study reported a nonadherence rate of 14.6%, and real-world nonadherence to adjuvant therapy may still be underestimated because of loss of patient follow-up and self-reporting methods [8].

### Treatment Noncompliance in Breast Cancer

#### Influence Factors of Noncompliance

Noncompliance is a complex and multifaceted phenomenon that is influenced by demographic, socioeconomic, psychological, and clinical-related factors [21-24]. Several studies including our study have documented that age-specific factors were associated with a lower compliance rate [17,25,26]. This was because weakness and fragility due to old age may influence tolerance to medical interventions. Regarding disease-related factors, receiving axillary lymph node dissection (ALND), HER2-positive, unfavorable molecular subtype, and advanced tumor, lymph node, and metastasis stage were associated with lower compliance. These patients usually require more complex and aggressive regimens, and their confusion about the therapeutic procedure as well as anxiety about adverse reactions may lead to treatment absence [27]. Moreover, the literature reported that health insurance and employment status were correlated with intravenous chemotherapy adherence [28,29]. Our study demonstrated that educational level was an influencing factor of compliance. These results indicate that economic level was positively related to treatment adherence. Jacobs et al [30] found that cancer-related symptom severity, but not sociodemographic or psychosocial constructs, could influence patients’ compliance with oral chemotherapy. The size of enrollment and the delivery method of chemotherapy may have caused differences between the studies. Furthermore, the modifiability of psychological status renders it a key to improving patient compliance [31], but we failed to include this factor in the study because of the difficulty in emotional evaluation during regular follow-up.

#### Interventions to Improve Patients’ Compliance

Withdrawal from treatment was associated with increased recurrence and impaired survival [32]. Our previous study showed that noncompliant patients had 1.8 times the risk of disease relapse and 2.5 times all-cause mortality than those who received the planned treatment [7]. Thus, tailoring enhanced interventions to improve patient adherence is of great importance. Liu et al [33] showed a large difference in patient adherence (59% vs 94%) between those receiving provider-patient communications and those not receiving provider-patient communication. In SWOG S1105, unidirectional text messaging failed to reduce the early discontinuation of adjuvant aromatase inhibitor therapy in women with early-stage breast cancer, suggesting that long-term adherence may call for personalized behavioral interventions and sustained management [34]. A meta-analysis by Lin et al [35] analyzed the influence of psychosocial factors on oral anticancer medication adherence in patients with breast cancer and indicated that using patient-centered interventions and building sustainable relationships may contribute to improved compliance.

### Mobile Health Apps

#### Purpose of Mobile Health Apps

During the treatment process, patients may face complex procedures, unfamiliar treatment-related effects, and numerous changes in psychology and lifestyle. Thus, they need a platform to interact with medical workers, provide feedback on treatment-related problems, and gain informational support. With the popularization of mobile communication, a number of mobile health apps designed for prevention and early diagnosis of disease, management of disease, and survival support and enhancement, greatly facilitate breast cancer health care. As acknowledged, the treatment of breast cancer involves more than surgery; thus, our app was designed for the management of postoperative treatment. Until 2018, approximately 600 apps were designed for breast cancer prevention and management [36]. Those apps designed for providing disease-related information, managing disease, raising awareness, and preventing disease, and the majority of the target population of these apps were patients with breast cancer.

#### Features and Innovations of Our App

We published an app that engages physicians and oncology nurses in the long-term management of patients after surgery. Our app relies on the most popular social networking platform in China-WeChat, and patients do not need to download and install the software, which ensures convenience and accessibility. Meanwhile, the app was connected to our MDT decision support system [37], and the treatment information of patients can be directly imported into the full-course management app system without manual operation, which could improve efficacy and reduce errors. More than half of the previous apps were developed by nonmedical professors, which may leave concerns regarding the accuracy and validity of the information [36,38]. Our app was designed by health care and computer science professionals, which ensured the credibility of the information and reliability of operation. Several studies have reported that mobile intervention only had modest effects on patients’ actual behavior, especially using the most common type of intervention-text messaging [34,39]. We found that after adjusting for the diagnosis year, patients using the smartphone-based full-course management app had a nonadherence rate of only 9.1%, lower than 17.6% in the Pre-App cohort, and 13.24% in the App nonused cohort. The effectiveness of our app may be because, in addition to regular treatment reminders, it supports interactive communication between patients and specialist nurses, which enables the patients to resolve the problem during treatment in a timely manner. The importance of two-way communication has also been demonstrated by Hwang et al [40], who designed an e-monitoring app allowing patients to upload the picture of wounds and consulting doctors on web, and it can decrease the number of clinical visits.

#### Patient Engagement

Patient engagement was expected to be related to the effectiveness of the digital intervention. Perski et al [41] found that the target behavior, as well as the mechanism of action, may influence engagement. At present, there is a lack of a uniform standard definition of engagement. In this study, we defined the use by logging detected from the backstage system for at least one time, as well as the reasons for patients not to use this app, including not owning a smartphone, older age, being treated in the local hospitals, and refusing to follow the MDT recommendation. Stubbins et al [42] emphasized the importance of providing real-time feedback via the app to improve patients’ engagement and adherence. Our app has a feedback function, tending to investigate and improve user experience. A previous review summarized studies using interventions to improve patients’ endocrine therapy drug uptake, which should last for years, and all published studies failed to prevent medication discontinuation [43]. Our app was also not effective for targeted or endocrine therapy. Indeed, with the prolongation of treatment, user retention for smartphone-based apps is another great challenge [44], and further efforts are still needed to minimize user fatigue to enhance patients’ long-term engagement.

#### Economic Factors

There has been growing interest in economic evidence. Although our app is funded by a government project and is completely free for the public to use, the economic status could still potentially influence the use of mobile communication. However, there is a lack of uniform standards to evaluate the economic outcomes, and cost-effectiveness analysis is still insufficient [45,46].

### Limitations

In this study, we developed a novel and effective smartphone-based app that can decrease patients’ prescription-deviating behaviors during treatment. One strength of this study is the large sample size of the included patients. There are several other potential limitations of this study. First, as a retrospective study, there may be a selection bias among patients. Thus, a prospectively designed study is warranted to validate the influence of this app on patient adherence and long-term survival. Second, there is still a lack of standard methods for evaluating adherence. Further efforts are needed to achieve consensus in the assessment of compliance, including time, frequency, and detection method. Third, the economic status of patients was difficult to assess in this study, which may have greatly influenced the results. Tailoring appropriate criteria to evaluate the financial situation of patients should be considered in the future. Last but not least, the follow-up time is still too short for some of the patients right now because they have not completed adjuvant endocrine therapy for at least 5 years, and the results of continued follow-up are worth expecting. There were also some points that deserve consideration in the development of health care–related apps. First, standardized quality measures for medical apps are lacking, and corresponding scales or rules should be developed. The apps should be comprehensively measured and rated by medical workers, software engineers, and users to improve the quality of apps. Second, the economic impact and long-term effectiveness of apps should be constantly tracked, and novel features warrant consideration to meet patients’ needs and ensure user engagement.

### Conclusions

We included a large number of patients with early-stage breast cancer within the MDT discussion and found that treatment adherence was independently associated with smartphone-based app use, which can serve as a useful intervention to improve patient compliance with MDT. Prospective studies are needed to validate the effectiveness of smartphone-based full-course management apps in improving patient outcomes to integrate this app into routine MDT clinical practice for breast cancer.

## Appendix

Multimedia Appendix 1 Supplementary tables.

## Abbreviations

ALND: axillary lymph node dissection
HER2: human epidermal growth factor receptor-2
MDT: multidisciplinary treatment
OR: odds ratio
