# Supplementary material for: A Smartphone-Based App to Improve Adjuvant Treatment Adherence to Multidisciplinary Decisions in Patients With Early-Stage Breast Cancer: Observational Study
Source: J Med Internet Res. 2021 Sep 16;23(9):e27576. doi: 10.2196/27576 (PMC8485191; doi:10.2196/27576)
Supplement: Multimedia Appendix 1 [file jmir_v23i9e27576_app1.docx]

**Table S1** Treatment adherence of adjuvant chemotherapy

|  | Adherence | Nonadherence | *P* value |
| --- | --- | --- | --- |
| Year |  |  | 0.084 |
| 2013.3-2014.5 | 488 (91.6) | 45 (8.4) |  |
| 2014.6-2015.5 | 519 (93.0) | 39 (7.0) |  |
| 2015.6-2016.5 | 607 (92.1) | 52 (7.9) |  |
| 2016.6-2017.5 | 741 (93.2) | 54 (6.8) |  |
| 2017.6-2018.5 | 864 (92.4) | 71 (7.6) |  |
| 2018.6-2019.5 | 946 (95.1) | 49 (4.9) |  |
| Age (years) |  |  | <0.001 |
| ≤50 | 1579 (95.0) | 83 (5.0) |  |
| 50-70 | 2150 (93.3) | 154 (6.7) |  |
| >70 | 436 (85.7) | 73 (14.3) |  |
| Educational level |  |  | 0.077 |
| Middle school or lower | 1542 (92.2) | 131 (7.8) |  |
| High school or higher | 2535 (93.6) | 174 (6.4) |  |
| Marriage status |  |  | 0.341 |
| Married | 4019 (93.1) | 296 (6.9) |  |
| Others | 146 (91.3) | 14 (8.8) |  |
| Menopausal status |  |  | <0.001 |
| Pre- | 1644 (95.1) | 84 (4.9) |  |
| Post- | 2521 (91.8) | 226 (8.2) |  |
| Benign breast disease history |  |  | 0.224 |
| Yes | 909 (94.0) | 58 (6.0) |  |
| No | 3256 (92.8) | 252 (7.2) |  |
| Malignant disease history |  |  | 0.208 |
| Yes | 190 (90.9) | 19 (9.1) |  |
| No | 3975 (93.2) | 291 (6.8) |  |
| Family history of breast cancer |  |  | 0.181 |
| Yes | 323 (95.0) | 17 (5.0) |  |
| No | 3842 (92.9) | 293 (7.1) |  |
| Comorbidity |  |  | 0.014 |
| Yes | 1623 (91.9) | 143 (8.1) |  |
| No | 2542 (93.8) | 167 (6.2) |  |
| Breast surgery |  |  | 0.158 |
| Breast conserving | 1498 (93.8) | 99 (6.2) |  |
| Mastectomy | 2667 (92.7) | 211 (7.3) |  |
| Axillary surgery |  |  | 0.707 |
| SLNB | 2734 (93.0) | 206 (7.0) |  |
| ALND | 1401 (93.3) | 100 (6.7) |  |
| Tumor size |  |  | 0.091 |
| ≤2cm | 2381 (93.7) | 161 (6.3) |  |
| >2cm | 1702 (92.3) | 141 (7.7) |  |
| Lymph node status |  |  | 0.618 |
| Negative | 2709 (93.3) | 196 (6.7) |  |
| Positive | 1425 (92.8) | 110 (7.2) |  |
| Pathological subtype |  |  | 0.001 |
| IDC | 3563 (92.6) | 285 (7.4) |  |
| Non-IDC | 602 (96.0) | 25 (4.0) |  |
| Tumor grade |  |  | <0.001 |
| I/II | 2116 (93.9) | 137 (6.1) |  |
| III | 1434 (91.1) | 140 (8.9) |  |
| Unknown | 615 (94.9) | 33 (5.1) |  |
| LVI |  |  | 0.788 |
| No | 3652 (93.0) | 274 (7.0) |  |
| Yes | 513 (93.4) | 36 (6.6) |  |
| ER status |  |  | <0.001 |
| Negative | 1002 (89.1) | 122 (10.9) |  |
| Positive | 3163 (94.4) | 188 (5.6) |  |
| PR status |  |  | <0.001 |
| Negative | 1467 (90.1) | 162 (9.9) |  |
| Positive | 2698 (94.8) | 148 (5.2) |  |
| HER2 status |  |  | 0.486 |
| Negative | 3202 (93.2) | 233 (6.8) |  |
| Positive | 963 (92.6) | 77 (7.4) |  |
| Ki-67 |  |  | 0.002 |
| <14% | 1327 (94.8) | 73 (5.2) |  |
| ≥14% | 2838 (92.3) | 237 (7.7) |  |
| Molecular subtype |  |  | <0.001 |
| Luminal-A like | 895 (97.0) | 28 (3.0) |  |
| Luminal-B like (HER2-negative) | 1781 (93.6) | 122 (6.4) |  |
| Luminal-B like (HER2-positive) | 502 (93.0) | 38 (7.0) |  |
| HER2 positive | 461 (92.2) | 39 (7.8) |  |
| Triple negative | 526 (86.4) | 83 (13.6) |  |
| TNM stage |  |  | 0.139 |
| Stage I | 1833 (93.8) | 121 (6.2) |  |
| Stage II | 1708 (92.9) | 131 (7.1) |  |
| Stage III | 528 (91.5) | 49 (8.5) |  |
| Group according to App usage |  |  | <0.001 |
| Pre-App cohort | 2732 (92.1) | 234 (7.9) |  |
| App nonused cohort | 808 (93.8) | 53 (6.2) |  |
| App used cohort | 625 (96.5) | 23 (3.5) |  |

Abbreviation: SLNB=sentinel lymph node biopsy, ALND=axillary lymph node dissection, IDC=invasive ductal carcinoma, LVI=lymphovascular invasion, ER=estrogen receptor, PR=progesterone receptor, HER2= human epidermal growth factor receptor 2.

**Table S2** Multivariate analysis of factor associated with nonadherence of adjuvant chemotherapy

|  | OR | 95% CI | *P* value |
| --- | --- | --- | --- |
| Age |  |  | <0.001 |
| 50-70 vs. ≤50 | 1.35 | 1.03-1.79 | 0.033 |
| >70 years vs. ≤50 | 3.70 | 2.62-5.22 | <0.001 |
| Menopausal status (Post- vs. Pre-) | 0.76 | 0.47-1.24 | 0.276 |
| Comorbidity (Yes vs. No) | 1.07 | 0.82-1.39 | 0.614 |
| Pathological subtype (Non-IDC vs. IDC) | 0.52 | 0.34-0.80 | 0.003 |
| Tumor grade |  |  | 0.296 |
| III vs. I/II | 1.07 | 0.81-1.41 | 0.635 |
| Unknown vs. I/II | 1.64 | 0.88-3.08 | 0.122 |
| ER status (Positive vs. negative) | / | / | / |
| PR status (Positive vs. negative) | 0.86 | 0.59-1.27 | 0.456 |
| Ki-67 index (≥14% vs. <14%) | 0.84 | 0.58-1.21 | 0.345 |
| Molecular subtype |  |  | <0.001 |
| Luminal-B like (HER2 negative) vs. Luminal-A like | 2.34 | 1.53-3.58 | <0.001 |
| Luminal-B like (HER2 positive) vs. Luminal-A like | 2.89 | 1.74-4.83 | <0.001 |
| HER2 positive vs. Luminal-A like | 3.03 | 1.82-5.04 | <0.001 |
| Triple negative vs. Luminal-A like | 5.49 | 3.50-8.60 | <0.001 |
| Group according to App usage |  |  | <0.001 |
| App nonused cohort vs. Pre-App cohort | 0.74 | 0.54-1.02 | 0.067 |
| App used cohort vs. Pre-App cohort | 0.41 | 0.27-0.65 | <0.001 |

Abbreviation: OR=Odds ratio, CI=confidence interval, IDC=invasive ductal carcinoma, PR=progesterone receptor, HER2= human epidermal growth factor receptor 2.

**Table S3** Treatment adherence of adjuvant radiotherapy

|  | Adherence | Nonadherence | *P* value |
| --- | --- | --- | --- |
| Year |  |  | 0.003 |
| 2013.3-2014.5 | 485 (91.0) | 48 (9.0) |  |
| 2014.6-2015.5 | 523 (93.7) | 35 (6.3) |  |
| 2015.6-2016.5 | 623 (94.5) | 36 (5.5) |  |
| 2016.6-2017.5 | 755 (95.0) | 40 (5.0) |  |
| 2017.6-2018.5 | 896 (95.8) | 39 (4.2) |  |
| 2018.6-2019.5 | 949 (95.4) | 46 (4.6) |  |
| Age (years) |  |  | <0.001 |
| ≤50 | 1603 (96.5) | 59 (3.5) |  |
| 50-70 | 2192 (95.1) | 112 (4.9) |  |
| >70 | 436 (85.7) | 73 (14.3) |  |
| Educational level |  |  | <0.001 |
| Middle school or lower | 1546 (92.4) | 127 (7.6) |  |
| High school or higher | 2601 (96.0) | 108 (4.0) |  |
| Marriage status |  |  | 1.000 |
| Married | 4079 (94.5) | 236 (5.5) |  |
| Others | 152 (95.0) | 8 (5.5) |  |
| Menopausal status |  |  | <0.001 |
| Pre- | 1664 (96.3) | 64 (3.7) |  |
| Post- | 2567 (93.4) | 180 (6.6) |  |
| Benign breast disease history |  |  | 0.174 |
| Yes | 923 (95.4) | 44 (4.6) |  |
| No | 3308 (94.3) | 200 (5.7) |  |
| Malignant disease history |  |  | 0.433 |
| Yes | 195 (93.3) | 14 (6.7) |  |
| No | 4036 (94.6) | 230 (5.4) |  |
| Family history of breast cancer |  |  | 0.062 |
| Yes | 329 (96.8) | 11 (3.2) |  |
| No | 3902 (94.4) | 233 (5.6) |  |
| Comorbidity |  |  | 0.018 |
| Yes | 1652 (93.5) | 114 (6.5) |  |
| No | 2579 (95.2) | 130 (4.8) |  |
| Breast surgery |  |  | 0.086 |
| Breast conserving | 1497 (93.7) | 100 (6.3) |  |
| Mastectomy | 2734 (95.0) | 144 (5.0) |  |
| Axillary surgery |  |  | <0.001 |
| SLNB | 2823 (96.0) | 117 (4.0) |  |
| ALND | 1381 (92.0) | 120 (8.0) |  |
| Tumor size |  |  | <0.001 |
| ≤2cm | 2434 (95.8) | 108 (4.2) |  |
| >2cm | 1712 (92.9) | 131 (7.1) |  |
| Lymph node status |  |  | <0.001 |
| Negative | 2795 (96.2) | 110 (3.8) |  |
| Positive | 1408 (91.7) | 127 (8.3) |  |
| Pathological subtype |  |  | 0.185 |
| IDC | 3631 (94.4) | 217 (5.6) |  |
| Non-IDC | 600 (95.7) | 27 (4.3) |  |
| Tumor grade |  |  | 0.012 |
| I/II | 2144 (95.2) | 109 (4.8) |  |
| III | 1467 (93.2) | 107 (6.8) |  |
| Unknown | 620 (95.7) | 28 (4.3) |  |
| LVI |  |  | 0.315 |
| No | 3717 (94.7) | 209 (5.3) |  |
| Yes | 514 (93.6) | 35 (6.4) |  |
| ER status |  |  | 0.255 |
| Negative | 1055 (93.9) | 69 (6.1) |  |
| Positive | 3176 (94.8) | 175 (5.2) |  |
| PR status |  |  | 0.029 |
| Negative | 1524 (93.6) | 105 (6.4) |  |
| Positive | 2707 (95.1) | 139 (4.9) |  |
| HER2 status |  |  | 0.349 |
| Negative | 3254 (94.7) | 181 (5.3) |  |
| Positive | 977 (93.9) | 63 (6.1) |  |
| Ki-67 |  |  | 0.013 |
| <14% | 1341 (95.8) | 59 (4.2) |  |
| ≥14% | 2890 (94.0) | 185 (6.0) |  |
| Molecular subtype |  |  | 0.117 |
| Luminal-A like | 889 (96.3) | 34 (3.7) |  |
| Luminal-B like (HER2-negative) | 1794 (94.3) | 109 (5.7) |  |
| Luminal-B like (HER2-positive) | 508 (94.1) | 32 (5.9) |  |
| HER2 positive | 469 (93.8) | 31 (6.2) |  |
| Triple negative | 571 (93.8) | 38 (6.2) |  |
| TNM stage |  |  | <0.001 |
| Stage I | 1896 (97.0) | 58 (3.0) |  |
| Stage II | 1712 (93.1) | 127 (6.9) |  |
| Stage III | 527 (91.3) | 50 (8.7) |  |
| Group according to App usage |  |  | 0.001 |
| Pre-App cohort | 2789 (94.0) | 177 (6.0) |  |
| App nonused cohort | 809 (94.0) | 52 (6.0) |  |
| App used cohort | 633 (97.7) | 15 (2.3) |  |

Abbreviation: SLNB=sentinel lymph node biopsy, ALND=axillary lymph node dissection, IDC=invasive ductal carcinoma, LVI=lymphovascular invasion, ER=estrogen receptor, PR=progesterone receptor, HER2= human epidermal growth factor receptor 2.

**Table S4** Multivariate analysis of factor associated with nonadherence of adjuvant radiotherapy

|  | OR | 95% CI | *P* value |
| --- | --- | --- | --- |
| Diagnosis year (per year) | 0.85 | 0.76-0.96 | 0.009 |
| Age |  |  | <0.001 |
| 50-70 vs. ≤50 | 1.21 | 0.86-1.69 | 0.271 |
| >70 years vs. ≤50 | 4.36 | 2.95-6.44 | <0.001 |
| Educational level (high school or high vs. middle school or lower) | 0.57 | 0.43-0.75 | <0.001 |
| Menopausal status (Post- vs. Pre-) | 1.05 | 0.59-1.85 | 0.878 |
| Comorbidity (Yes vs. No) | 0.97 | 0.71-1.32 | 0.821 |
| Axillary surgery (ALND vs. SLNB) | 0.91 | 0.59-1.39 | 0.656 |
| Tumor grade |  |  | 0.229 |
| III vs. I/II | 1.23 | 0.89-1.70 | 0.210 |
| Unknown vs. I/II | 0.82 | 0.51-1.31 | 0.408 |
| PR status (positive vs. negative) | 0.88 | 0.65-1.20 | 0.410 |
| Ki-67 index (≥14% vs. <14%) | 1.65 | 1.17-2.33 | 0.004 |
| Tumor size (>2cm vs. ≤2cm) | 0.96 | 0.64-1.43 | 0.827 |
| Lymph node status (positive vs. negative) | 1.75 | 1.21-2.54 | 0.003 |
| TNM stage |  |  | 0.014 |
| Stage II vs. stage I | 1.78 | 1.21-2.62 | 0.004 |
| Stage III vs. stage I | 1.70 | 0.98-2.93 | 0.058 |
| Group according to App usage |  |  | 0.005 |
| App nonused cohort vs. Pre-App cohort | 1.38 | 0.86-2.22 | 0.180 |
| App used cohort vs. Pre-App cohort | 0.49 | 0.25-0.96 | 0.038 |

Abbreviation: OR=Odds ratio, CI=confidence interval, SLNB=sentinel lymph node biopsy, ALND=axillary lymph node dissection, PR=progesterone receptor, HER2= human epidermal growth factor receptor 2.

**Table S5** Treatment adherence of target therapy in HER2-positive breast cancer

|  | Adherence | Nonadherence | *P* value |
| --- | --- | --- | --- |
| Year |  |  | <0.001 |
| 2013.3-2014.5 | 85 (78.0) | 24 (22.0) |  |
| 2014.6-2015.5 | 105 (86.8) | 16 (13.2) |  |
| 2015.6-2016.5 | 143 (87.2) | 21 (12.8) |  |
| 2016.6-2017.5 | 185 (90.7) | 19 (9.3) |  |
| 2017.6-2018.5 | 180 (87.8) | 25 (12.2) |  |
| 2018.6-2019.5 | 224 (94.5) | 13 (5.5) |  |
| Age (years) |  |  | <0.001 |
| ≤50 | 368 (90.4) | 39 (9.6) |  |
| 50-70 | 516 (89.1) | 63 (10.9) |  |
| >70 | 38 (70.4) | 16 (29.6) |  |
| Educational level |  |  | 0.001 |
| Middle school or lower | 341 (84.4) | 63 (15.6) |  |
| High school or higher | 562 (91.4) | 53 (8.6) |  |
| Marriage status |  |  | 1.000 |
| Married | 896 (88.6) | 115 (11.4) |  |
| Others | 26 (89.7) | 3 (10.3) |  |
| Menopausal status |  |  | 0.037 |
| Pre- | 382 (91.2) | 37 (8.8) |  |
| Post- | 540 (87.0) | 81 (13.0) |  |
| Benign breast disease history |  |  | 0.720 |
| Yes | 195 (89.4) | 23 (10.6) |  |
| No | 727 (88.4) | 95 (11.6) |  |
| Malignant disease history |  |  | 0.464 |
| Yes | 36 (85.7) | 6 (14.3) |  |
| No | 886 (88.8) | 112 (11.2) |  |
| Family history of breast cancer |  |  | 0.356 |
| Yes | 72 (92.3) | 6 (7.7) |  |
| No | 850 (88.4) | 112 (11.6) |  |
| Comorbidity |  |  | 0.078 |
| Yes | 298 (86.1) | 48 (13.9) |  |
| No | 624 (89.9) | 70 (10.1) |  |
| Breast surgery |  |  | 0.635 |
| Breast conserving | 200 (89.7) | 23 (10.3) |  |
| Mastectomy | 722 (88.4) | 95 (11.6) |  |
| Axillary surgery |  |  | 0.165 |
| SLNB | 531 (90.0) | 59 (10.0) |  |
| ALND | 390 (87.2) | 57 (12.8) |  |
| Tumor size |  |  | 0.040 |
| ≤2cm | 448 (90.7) | 46 (9.3) |  |
| >2cm | 458 (86.6) | 71 (13.4) |  |
| Lymph node status |  |  | 0.619 |
| Negative | 538 (89.2) | 65 (10.8) |  |
| Positive | 383 (88.2) | 51 (11.8) |  |
| Pathological subtype |  |  | 0.536 |
| IDC | 865 (88.4) | 113 (11.6) |  |
| Non-IDC | 57 (91.9) | 5 (8.1) |  |
| Tumor grade |  |  | 0.127 |
| I/II | 344 (91.0) | 34 (9.0) |  |
| III | 519 (86.9) | 78 (13.1) |  |
| Unknown | 59 (90.8) | 6 (9.2) |  |
| LVI |  |  | 0.898 |
| No | 758 (88.6) | 98 (11.4) |  |
| Yes | 164 (89.1) | 20 (10.9) |  |
| ER status |  |  | 0.846 |
| Negative | 447 (88.9) | 56 (11.1) |  |
| Positive | 475 (88.5) | 62 (11.5) |  |
| PR status |  |  | 0.264 |
| Negative | 583 (87.8) | 81 (12.2) |  |
| Positive | 339 (90.2) | 37 (9.8) |  |
| Ki-67 |  |  | 0.237 |
| <14% | 80 (85.1) | 14 (14.9) |  |
| ≥14% | 842 (89.0) | 104 (11.0) |  |
| Molecular subtype |  |  | 0.770 |
| Luminal-B like (HER2-positive) | 477 (88.3) | 63 (11.7) |  |
| HER2 positive | 445 (89.0) | 55 (11.0) |  |
| TNM stage |  |  | 0.280 |
| Stage I | 315 (90.8) | 32 (9.2) |  |
| Stage II | 416 (87.4) | 60 (12.6) |  |
| Stage III | 175 (87.5) | 25 (12.5) |  |
| Group according to App usage |  |  | 0.013 |
| Pre-App cohort | 594 (86.6) | 92 (13.4) |  |
| App nonused cohort | 158 (91.9) | 14 (8.1) |  |
| App used cohort | 170 (93.4) | 12 (6.6) |  |

Abbreviation: SLNB=sentinel lymph node biopsy, ALND=axillary lymph node dissection, IDC=invasive ductal carcinoma, LVI=lymphovascular invasion, ER=estrogen receptor, PR=progesterone receptor, HER2= human epidermal growth factor receptor 2.

**Table S6** Multivariate analysis of factor associated with nonadherence of target therapy in HER2-positive breast cancer patients

|  | OR | 95% CI | *P* value |
| --- | --- | --- | --- |
| Diagnosis year (per year) | 0.77 | 0.68-0.87 | <0.001 |
| Age |  |  | <0.001 |
| 50-70 vs. ≤50 | 1.01 | 0.65-1.56 | 0.981 |
| >70 years vs. ≤50 | 4.17 | 2.07-8.40 | <0.001 |
| Educational level (high school or high vs. middle school or lower) | 0.51 | 0.34-0.76 | 0.001 |
| Menopausal status (Post- vs. Pre-) | 0.72 | 0.36-1.44 | 0.359 |
| Tumor size (>2cm vs. ≤2cm) | 1.48 | 0.98-2.23 | 0.060 |
| Group according to App usage |  |  | 0.933 |
| App nonused cohort vs. Pre-App cohort | 0.88 | 0.40-1.92 | 0.742 |
| App used cohort vs. Pre-App cohort | 0.88 | 0.39-1.98 | 0.756 |

Abbreviation: OR=Odds ratio, CI=confidence interval.

**Table S7** Treatment adherence of endocrine therapy in hormone receptor-positive breast cancer

|  | Adherence | Nonadherence | *P* value |
| --- | --- | --- | --- |
| Year |  |  | <0.001 |
| 2013.3-2014.5 | 344 (88.2) | 46 (11.8) |  |
| 2014.6-2015.5 | 363 (88.8) | 46 (11.2) |  |
| 2015.6-2016.5 | 443 (91.9) | 39 (8.1) |  |
| 2016.6-2017.5 | 550 (93.1) | 41 (6.9) |  |
| 2017.6-2018.5 | 692 (93.0) | 52 (7.0) |  |
| 2018.6-2019.5 | 722 (95.8) | 32 (4.2) |  |
| Age (years) |  |  | <0.001 |
| ≤50 | 1181 (92.3) | 98 (7.7) |  |
| 50-70 | 1574 (94.0) | 101 (6.0) |  |
| >70 | 359 (86.3) | 57 (13.7) |  |
| Educational level |  |  | 0.089 |
| Middle school or lower | 1104 (91.3) | 105 (8.7) |  |
| High school or higher | 1940 (93.0) | 147 (7.0) |  |
| Marriage status |  |  | 0.485 |
| Married | 3004 (92.5) | 245 (7.5) |  |
| Others | 110 (90.9) | 11 (9.1) |  |
| Menopausal status |  |  | 0.596 |
| Pre- | 1247 (92.1) | 107 (7.9) |  |
| Post- | 1867 (92.6) | 149 (7.4) |  |
| Benign breast disease history |  |  | 0.206 |
| Yes | 683 (93.6) | 47 (6.4) |  |
| No | 2431 (92.1) | 209 (7.9) |  |
| Malignant disease history |  |  | 0.212 |
| Yes | 139 (89.7) | 16 (10.3) |  |
| No | 2975 (92.5) | 240 (7.5) |  |
| Family history of breast cancer |  |  | 0.025 |
| Yes | 240 (96.0) | 10 (0.6) |  |
| No | 2874 (92.1) | 246 (7.9) |  |
| Comorbidity |  |  | 0.290 |
| Yes | 1280 (93.0) | 96 (7.0) |  |
| No | 1834 (92.0) | 160 (8.0) |  |
| Breast surgery |  |  | 0.459 |
| Breast conserving | 1159 (92.9) | 89 (7.1) |  |
| Mastectomy | 1955 (92.1) | 167 (7.9) |  |
| Axillary surgery |  |  | 0.017 |
| SLNB | 2098 (93.3) | 150 (6.7) |  |
| ALND | 997 (91.0) | 99 (9.0) |  |
| Tumor size |  |  | 0.007 |
| ≤2cm | 1888 (93.5) | 131 (6.5) |  |
| >2cm | 1167 (90.9) | 117 (9.1) |  |
| Lymph node status |  |  | <0.001 |
| Negative | 2051 (93.8) | 136 (6.2) |  |
| Positive | 1043 (90.2) | 113 (9.8) |  |
| Pathological subtype |  |  | 0.275 |
| IDC | 2641 (92.2) | 224 (7.8) |  |
| Non-IDC | 473 (93.7) | 32 (6.3) |  |
| Tumor grade |  |  | 0.001 |
| I/II | 1854 (93.4) | 130 (6.6) |  |
| III | 763 (9.4) | 90 (10.6) |  |
| Unknown | 497 (93.2) | 36 (6.8) |  |
| LVI |  |  | 0.549 |
| No | 2742 (92.5) | 222 (7.5) |  |
| Yes | 372 (91.6) | 34 (8.4) |  |
| ER status |  |  | 0.171 |
| Negative | 16 (84.2) | 3 (15.8) |  |
| Positive | 3098 (92.5) | 253 (7.5) |  |
| PR status |  |  | 0.151 |
| Negative | 476 (90.8) | 48 (9.2) |  |
| Positive | 2638 (92.7) | 208 (7.3) |  |
| HER2 status |  |  | 0.005 |
| Negative | 2628 (93.0) | 198 (7.0) |  |
| Positive | 486 (89.3) | 58 (20.7) |  |
| Ki-67 |  |  | 0.003 |
| <14% | 1208 (94.2) | 75 (5.8) |  |
| ≥14% | 1906 (91.3) | 181 (8.7) |  |
| Molecular subtype |  |  | <0.001 |
| Luminal-A like | 877 (95.0) | 46 (5.0) |  |
| Luminal-B like (HER2-negative) | 1751 (92.0) | 152 (8.0) |  |
| Luminal-B like (HER2-positive) | 483 (89.4) | 57 (10.6) |  |
| TNM stage |  |  | <0.001 |
| Stage I | 1473 (94.4) | 88 (5.6) |  |
| Stage II | 1195 (91.3) | 114 (8.7) |  |
| Stage III | 376 (89.3) | 45 (10.7) |  |
| Group according to App usage |  |  | <0.001 |
| Pre-App cohort | 2001 (91.2) | 194 (8.8) |  |
| App nonused cohort | 637 (93.5) | 44 (6.5) |  |
| App used cohort | 476 (96.4) | 18 (3.6) |  |

Abbreviation: SLNB=sentinel lymph node biopsy, ALND=axillary lymph node dissection, IDC=invasive ductal carcinoma, LVI=lymphovascular invasion, ER=estrogen receptor, PR=progesterone receptor, HER2= human epidermal growth factor receptor 2.

**Table S8** Multivariate analysis of factor associated with nonadherence of endocrine therapy in hormone receptor-positive breast cancer patients

|  | OR | 95% CI | P value |
| --- | --- | --- | --- |
| Diagnosis year (per year) | 0.84 | 0.72-0.99 | 0.032 |
| Age |  |  | 0.013 |
| 50-70 vs. ≤50 | 1.09 | 0.61-1.92 | 0.780 |
| >70 years vs. ≤50 | 3.41 | 1.46-7.99 | 0.005 |
| Family history of breast cancer (yes vs. no) | 0.54 | 0.16-1.85 | 0.323 |
| Axillary surgery (ALND vs. SLNB) | 1.18 | 0.68-2.06 | 0.554 |
| Tumor grade |  |  | 0.353 |
| III vs. I/II | 1.36 | 0.77-2.40 | 0.297 |
| Unknown vs. I/II | 0.65 | 0.20-2.12 | 0.479 |
| HER2 status (Positive vs. negative) | / | / | / |
| Ki-67 index (≥14% vs. <14%) | 0.82 | 0.31-2.18 | 0.697 |
| Molecular subtype |  |  | <0.001 |
| Luminal-B like (HER2 negative) vs. Luminal-A like | 0.55 | 0.03-8.80 | 0.671 |
| Luminal-B like (HER2 positive) vs. Luminal-A like | 156.80 | 21.31-1153.87 | <0.001 |
| Tumor size (>2cm vs. ≤2cm) | 1.56 | 0.91-2.69 | 0.105 |
| Lymph node status (positive vs. negative) | 0.75 | 0.30-1.87 | 0.541 |
| TNM stage |  |  | 0.832 |
| Stage II vs. stage I | 1.43 | 0.45-4.55 | 0.548 |
| Stage III vs. stage I | 1.54 | 0.33-7.26 | 0.583 |
| Group according to App usage |  |  | 0.543 |
| App nonused cohort vs. Pre-App cohort | 1.07 | 0.41-2.79 | 0.893 |
| App used cohort vs. Pre-App cohort | 0.63 | 0.22-1.80 | 0.393 |

Abbreviation: OR=Odds ratio, CI=confidence interval, SLNB=sentinel lymph node biopsy, ALND=axillary lymph node dissection, HER2= human epidermal growth factor receptor 2.
